# Calibration of Ultrasonic Transducer Based on Ultrasonic Logging Instrument for Shaft Sinking

**DOI:** 10.3390/s22186867

**Published:** 2022-09-11

**Authors:** Maoyong Cao, Xiaoan Si, Hui Zhang, Fengying Ma, Peng Ji, Hui Yao

**Affiliations:** School of Information and Automation Engineering, Qilu University of Technology (Shandong Academy of Sciences), Jinan 250300, China

**Keywords:** high-precision logging equipment, ultrasonic ranging, ultrasonic transducer calibration, ultrasonic transducer error

## Abstract

High-precision logging equipment is critical for measuring the borehole diameter and drilling offset in coal mining and petroleum drilling. We propose a module composition and positioning principle for an ultrasonic transducer based on an ultrasonic logging instrument for shaft sinking by drilling (ULISSD) for calculating the reflection distance. The logging distance, which is the primary performance index of a logging system, is determined by using the self-reception sensitivity and error of the ultrasonic transducer in a downhole system. To measure the error between the piezoelectric element of the transducer and the rubber seal of the borehole logging system, we developed an ultrasonic-transducer error-calibration device and a calibration method for a central-air-return-shaft-drilling project. This calibration device can eliminate the inherent error of the transducer and calculate the rate of propagation with high accuracy. The measurement error is reduced by approximately 1.5 mm; thus, the ULISSD measurement accuracy can be effectively improved in central-air-return-shaft drilling.

## 1. Introduction

In drilling applications in the coal and nuclear industries and basic engineering applications, it is necessary to correctly detect the size and deviation of drilling deflection, the degree of enlargement or reduction of the diameter and depth position, and the 3D shape of the borehole. Otherwise, if the drilling error is large, the drilling will be scrapped, thereby wasting manpower and material resources. Compared with the currently used ultrasonic logging tools, an ultrasonic logging instrument calibrated with a transducer reduces the transducer and sound velocity errors in an actual test environment, thereby enabling more accurate caliper and azimuth measurements [1,2,3,4]. Currently, an ultrasonic transducer calibration method [5,6,7] consumes substantial time and energy and requires recalibration in a new environment. The proposed ultrasonic transducer calibration method in this study has the advantages of simple calibration, less calibration time, and low calibration cost.

An ultrasonic transducer converts the input electrical energy into mechanical energy (i.e., ultrasound), which is then transmitted with low power consumption. The input section is driven by a sinusoidal voltage signal that generates vibrations through an inverse piezoelectric effect. The vibration wave is mechanically coupled to the output through the input, and the output section generates an electrical charge through a positive piezoelectric effect. Two transformations of piezoelectric mechanical and electrical energy are realized, and the highest output voltage is obtained at the resonant frequency of the piezoelectric transformer.

Calibration testing plays an essential role in industrial measurements. A calibration is a direct tool, an important method, and a key resource for laboratory inspection and testing activities. It is also an important component for enhancing the technical capabilities of a laboratory and a prerequisite for implementing laboratory quality policy and objectives. China’s accreditation criteria state that “the laboratory shall be equipped with all sampling, measurement, and testing equipment necessary for proper testing and calibration, including sampling, item preparation, data processing, and analysis”. Therefore, a laboratory should be equipped with appropriate testing equipment according to the content and scope of the test items that can be operated after verification or calibration.

Currently, large-caliber ultrasonic logging tools are widely used in the coal mining and drilling industry. The challenges associated with ultrasonic logging equipment in ultrasonic-transducer calibration have attracted the attention of researchers. Solving these challenges is the key to improving the accuracy and reliability of logging equipment and thereby reducing the errors and cost of ultrasonic-drilling and logging-tool ultrasonic-drilling calibrations. Time-delay spectroscopy, optical interference, and adaptive temperature calibration have previously been proposed for ultrasonic-transducer calibration [8,9,10].

The difference between the calibration method proposed in this paper and the transmission method [11] is that the penetration method requires two ultrasonic transducers, one to transmit ultrasound and the other to receive ultrasound; this requires the placement of two ultrasonic transducers to occupy a large experimental space and to ensure the coaxiality of the two ultrasonic transducers, which is difficult to achieve in a manual manner, so automated mechanical movement is often used instead of manual operation. However, the calibration method in this study used only an ultrasonic transducer that could be self-generated and self-accepted to conduct experiments that took up little experimental space and did not need to consider the coaxial problem of the ultrasonic transducer and the reflecting surface.

**Figure 1:**
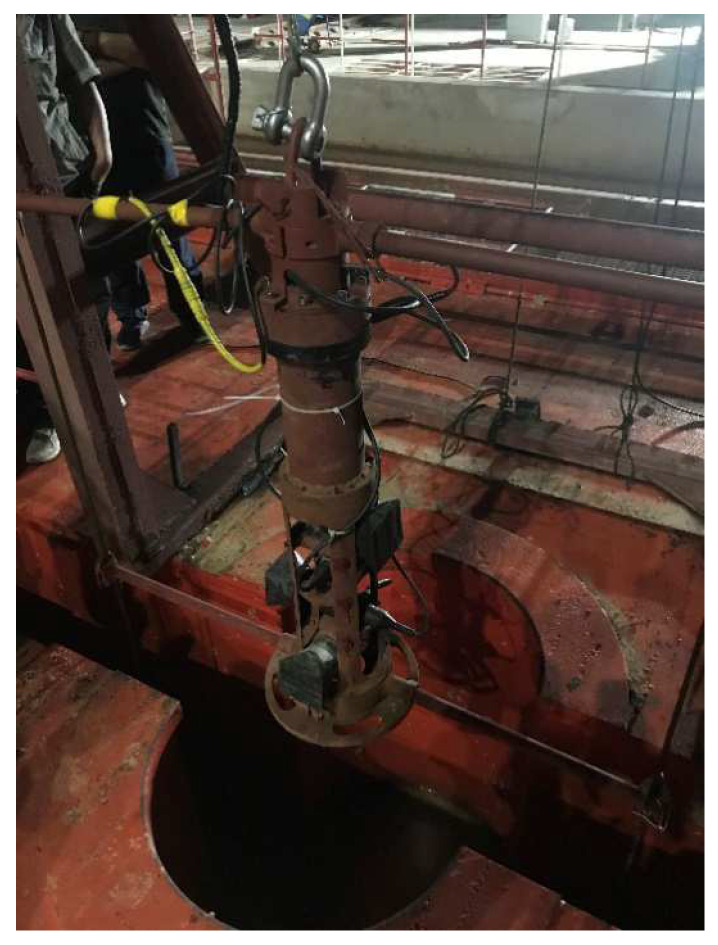
ULISSD used to log the central-air-return shaft.

China Coal Special Drilling Co., Ltd. (Yulin City, Shanxi Province, China), used the downhole ultrasonic logging instrument for shaft sinking by drilling (ULISSD) [1] to measure the first central-air-return vertical-shaft-drilling project in China via the drilling method shown in Figure 1. It pioneered the construction of this drilling method in the water-rich soft-rock area in Western China. When recording a central-air-return shaft with the ULISSD, the irregular shape of the shallow surface of the wellhead leads to a significant measurement error in the measurement data. The error gradually decreases when the depth drops to a certain level. Because of the limitations and complexity of the calibration methods proposed in References [8,12,13,14,15,16], the currently used ultrasonic logging equipment often does not calibrate transducers appropriately when solving the error of the ULISSD in the logging process of the central-air-return shaft. For example, a transducer emits ultrasonic waves by default and receives echoes for the piezoelectric element on the rubber surface when the logging equipment is used in the range. Uncalibrated transducers are limited to measuring the general diameter and azimuth deviation of coal mines. As measurement-accuracy requirements increase, the calibration of the transducers becomes important. No calibration scheme has been proposed for the use of the existing ultrasonic logging instrument transducers.

In this study, a device for calibrating ultrasonic transducers and a fixed-error calibration method were developed to calculate the fixed error of an ultrasonic transducer. An ultrasonic sensor was controlled by a translational slider on the ultrasonic-sensor calibration device to estimate the distance from the reflecting surface. The distance between the ultrasonic transducer and the reflecting surface could be adjusted manually throughout the test. To improve the accuracy of the adjustment distance, the controller could also be used to control the sliding device to automatically adjust to the set fixed distance. Through multiple experimental measurements, the measured experimental data were calculated and analyzed to obtain the fixed error of the ultrasonic transducer. This method was then applied to the ULISSD.

The rest of the paper is organized as follows. Section 2 describes the principle of ultrasonic positioning, introduces the method for ultrasonic transducer calibration, and describes the design of the ultrasonic-transducer calibration device. Section 3 presents an experimental application of the ultrasonic-transducer calibration device and the corresponding experimental evaluation. Finally, Section 4 presents the conclusions.

## 2. Materials and Methods

### 2.1. Development Status of Ultrasonic Transducer Calibration Methods

Currently, the proposed calibration methods include the laser interferometer ultrasonic-transducer calibration and adaptive-temperature calibration. The former can calibrate ultrasonic transducers based on sensor performance evaluation in a variety of environments, geometries, and material types. Javier successfully developed a portable laser interferometer that has sufficient sensitivity to be used as an ultrasonic detector in laboratories and also in industrial environments.

Most existing ultrasonic-transducer calibration methods are only theoretical. In other words, no actual experiment has been conducted to verify their feasibility. Additionally, most of the realized calibration methods are only suitable for high-frequency ultrasonic transducers, whereas the calibration method proposed in this study is suitable for both high- and low-frequency ultrasonic transducers simultaneously. Finally, compared with other methods, the proposed calibration method is simple to realize, does not need multiple calibrations, has strong practicability, and is low in cost.

### 2.2. Principle of Ultrasonic Positioning

When calculating the parameters from the drilling center to the borehole wall [17,18,19], two sets of values are needed: the distance from the center of the borehole to the wall and the vertical distance from the measurement point. In this study, a field calibration method was primarily used to correct the error of the ULISSD in terms of the calculated distance from the center of the borehole to the borehole wall, thereby improving the accuracy of the measured distance.

A microcontroller in the distance measurement module of the wellhead operates in a timing mode. After a trigger circuit receives a trigger signal, a single-chip microcomputer starts timing the ultrasonic signal. In the next step, when the transducer receives an echo signal, it stops timing and reads the values of the counter, which are the original data. In the upper computer software, the original data are first processed to determine the well-diameter value. The parameter Δ*t* is determined by using the following equation:

(1) $$\Delta t=m\times \frac{12}{T_{osc}}$$

where *T_osc_* is the crystal oscillation frequency of a single-chip microcomputer, and *m* is the counting rate of the machine cycles of the single-chip microcomputer. The constant 12 appears because the equipment using a single-chip microcomputer machine cycle consists of 12 clock cycles. The distance between the transducer and shaft wall, *L*, can then be calculated by using Equation (2), where *v* is the sound velocity:

(2) $$L=\frac{1}{2}v\Delta t$$

### 2.3. Errors in the Ultrasonic Positioning System

The measurement errors of ultrasonic transducers are primarily caused by the interference of the medium and shortcomings of the device design [20,21].

The following quantitative analysis factors can affect the positioning error. First, in the actual measurement, the sound velocity, *v*, is not constant and is greatly influenced by changes in the medium; the magnitude of *v* in mud; and the mud parameters, such as the temperature and concentration. Therefore, the sound velocity of the ULISSD in mud cannot easily be determined.

Second, an ultrasonic transducer is composed of piezoelectric materials, which are fragile. Because of the various requirements of insulation, such as sealing, corrosion resistance, impedance matching, and other factors, piezoelectric elements are usually installed in a rubber shell, which becomes the probe. The piezoelectric material and the surface of the probe are separated by a distance, *D*_0_. Owing to the different manufacturing variability, the *D*_0_ for different transducers may vary, thereby resulting in measurement errors. Therefore, before using the probe, the distance between the piezoelectric plate of the probe and the surface of the probe should be calculated to eliminate these errors. Because a finished probe cannot be disassembled for direct measurement, only indirect measurements are performed. Therefore, field calibration is required to eliminate these errors [22,23,24].

**Figure 2:**
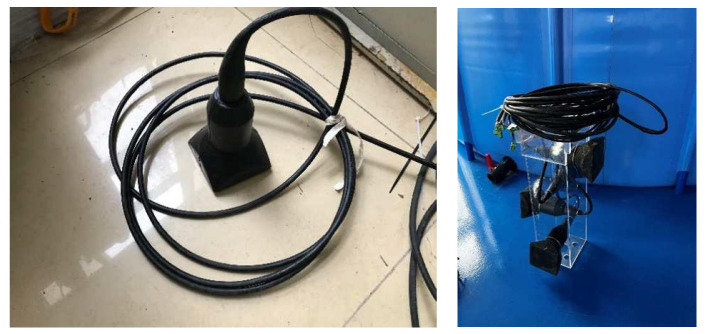
Proposed ultrasonic transducer.

The ultrasonic transducer used in this paper is shown in Figure 2, and the ultrasonic transducer used in this paper was made with a frequency of 10 kHz and a diameter of 8 centimeters, using The Qingdao Branch and North Sea Research Station, Institute of Acoustics, Chinese Academy of Sciences.

### 2.4. Calibration Principle of the Ultrasonic Transducer

**Figure 3:**
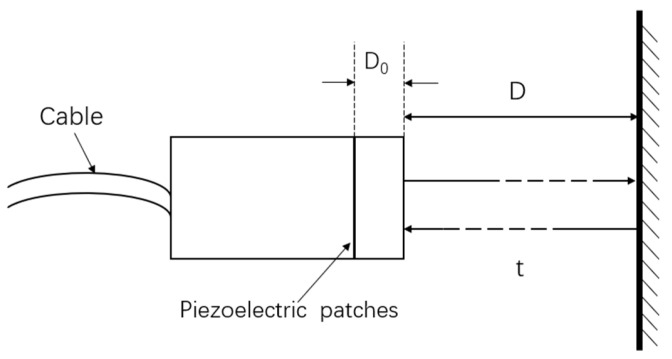
Schematic of the error calculation from the ultrasonic transducer to the borehole wall.

The known fixed distances from the probe surface to the well walls are *D*_1_ and *D*_2_. Moreover, *t*_1_ and *t*_2_ correspond to the time taken for the ultrasonic probe to receive an echo at two fixed distances [25,26], as shown in Figure 3.

Through theoretical derivation, we have the following:

(3) $$\{\begin{array}{c} D_{1}+D_{0}=\frac{1}{2}v_{0}t_{0}+\frac{1}{2}v\left(t_{1}-t_{0}\right)\\ D_{2}+D_{0}=\frac{1}{2}v_{0}t_{0}+\frac{1}{2}v\left(t_{2}-t_{0}\right) \end{array}$$

where *v*_0_ is the speed of sound of the ultrasonic wave in the rubber, and *t*_0_ is the time it takes for the ultrasonic wave to pass through the rubber.

According to Equation (3), we can obtain the following:

(4) $$D_{0}=\frac{D_{2}-D_{1}}{t_{2}-t_{1}}\cdot t_{1}-D_{1}$$

In Equation (4), the piezoelectric distance, *D*_0_, is proportional to the probe work error.

**Figure 4:**
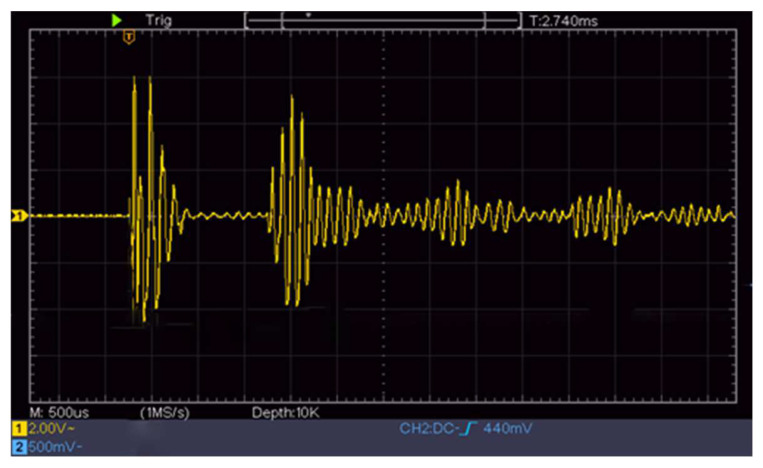
Ultrasonic echo time.

The transmitted signal and received echo signal of the ultrasonic transducer are shown in Figure 4, where time, *t*, is the elapsed time from the transmitted signal to the receipt of the first echo signal.

Figure 4 shows a screenshot of the oscilloscope measurement of the echo signal received by the ultrasonic transducer, where the *x*-axis is set at 500 ms per frame, and the *y*-axis is set at 2 V per frame.

### 2.5. Design of Ultrasonic-Transducer Calibration Device

To apply the abovementioned theoretical considerations in practice, a probe-error-correction experiment was conducted in the ultrasonic-logging-instrument laboratory of the Research Institute of Detection and Control of Qilu University of Technology (Shandong Academy of Sciences, Jinan, Shandong, China). First, a transducer calibration device was developed to facilitate the measurement of the required values.

The primary components of the ultrasonic-transducer calibration device (longitudinal) are an outer frame built of profiles, an interior composed of a container measuring 1200–150 mm high and filled with the required medium for the experiment, and an ultrasonic transducer that can perform self-generation and self-acceptance. The profile frame’s height extends beyond the height of the bucket and defines the longitudinal translation axis (*y*-axis). Each coordinate positioning system consists of a vertical (*y*-axis) translation slider, which supports the ultrasonic transducer and provides adjustment about the *y*-axis, as the transducer is fixed on a bracket secured to the vertical bar.

**Figure 5:**
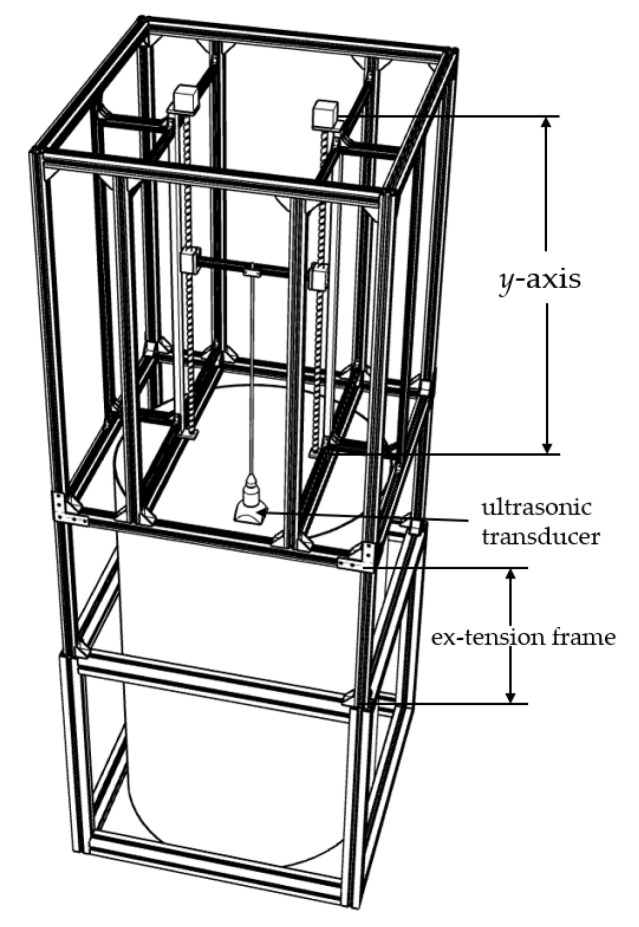
Longitudinal-ultrasonic-transducer calibration device with a height of 1200~1500 mm.

While designing the ultrasonic-transducer calibration device (longitudinal), an extension frame with an adjustable height was designed to insert a higher bucket in the profile frame and thus improve the measurement distance during calibration. As shown in Figure 5, the extended ultrasonic-transducer calibration device (longitudinal) can hold a measurement container with a maximum height of 1500 mm. Therefore, the variable range of the distance of the ultrasonic transducer was improved, and more experimental data could be provided.

The ultrasonic-transducer calibration device (transverse) comprises an outer frame of profiles that is 3500 mm wide when mounted and 1200 mm high, a circular pool with a diameter of 3000 mm, and two ultrasonic transducers that are capable of self-collection. The profile extends beyond the pool diameter distance and determines the transverse translation axis (*x*-axis). Each coordinate positioning system consists of horizontal translational sliders to accommodate the two ultrasonic transducers. To set the *y*-axis adjustment, the ultrasonic transducers are attached to a horizontal rod support.

All the movements can be adjusted manually. However, to improve the efficiency of the experiment and the accuracy of the positional movement of the devices, each experimental device is equipped with two stepper motors controlled by a microcontroller. The corresponding movement of 0.05 mm for each step is the translation stage. In normal usage, only the translation of the ultrasonic transducer is controlled by the microcontroller.

### 2.6. Composition and Realization of Ultrasonic-Transducer Calibration Platform

Based on the abovementioned designs and methods, two sets of longitudinal and transverse platforms were built to correct the errors of the ultrasonic transducers. The longitudinal calibration platform comprised a longitudinal-ultrasonic-transducer calibration device, an ultrasonic transducer, a time-data acquisition module based on STM32, and a computer system.

**Figure 6:**
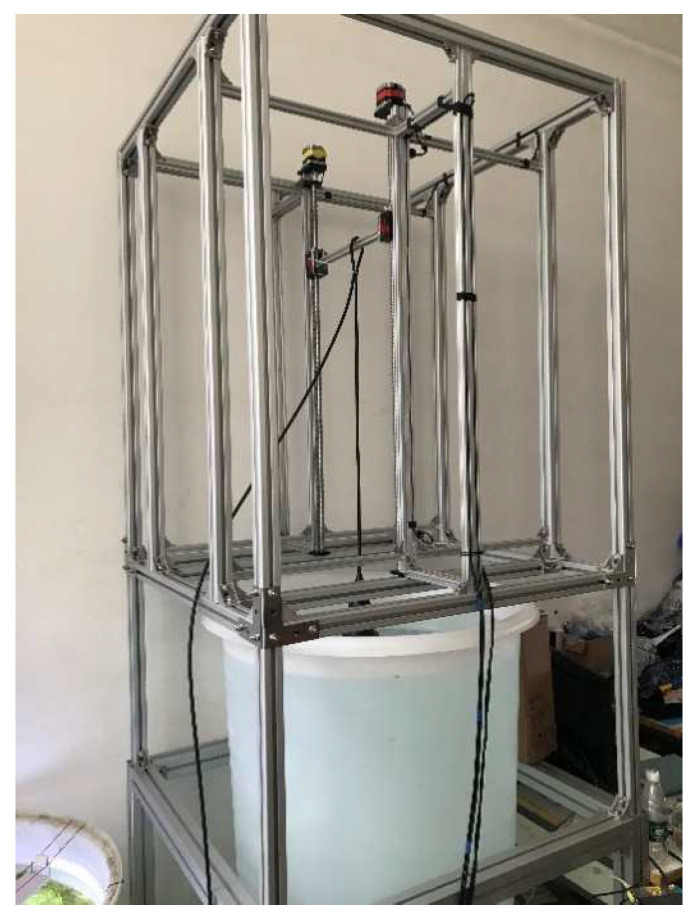
Longitudinal-ultrasonic-transducer error-calibration-test platform with a height of 1500 mm.

The longitudinal calibration platform was extended to a height of 1500 mm, as shown in Figure 6. The calibration device was first placed on a suitably wide plastic bucket with a height of 1200 mm to simulate the adjustable distance between the probe and the well wall. By increasing the height of the calibration device, a taller bucket (1500 mm) could be placed in the calibration device to improve the threshold of the simulated distance. After repeated measurements of the transducer, it was concluded that the transverse calibration platform was suitable for the calibration of high-frequency ultrasonic transducers, owing to the small measurable distance.

The longitudinal-error-correction platform parameters are listed in Table 1.

### 2.7. Operating Principle of the Ultrasonic Transducer Calibration Device

**Figure 7:**
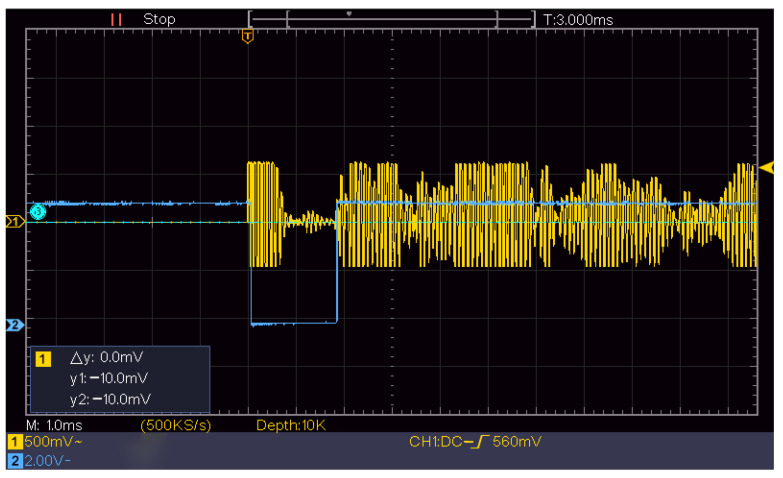
Echo of the probe at 1300 mm recorded by an oscilloscope.

The initial height of the experiment was 1320 mm. At 1300 mm, the ultrasonic-transducer signal received by the ULISSD was recorded by an oscilloscope, as shown in Figure 7. The attenuating signal in the first half was the spontaneous signal from the ultrasonic transducer, and the signal in the second half was the first echo signal received by the transducer. The time-data acquisition module STM32 was used to calculate the time from the transmitted signal to the reception of the echo signal.

Figure 7 shows a screenshot of the oscilloscope measurement of the echo signal received by the ultrasonic transducer, where the *x*-axis is set at 1 ms per frame, and the *y*-axis is set at 500 mV per frame.

When the devices shown in Figure 6 were used for calibration, the diameter of the container placed in the calibration platform of the ULISSD with a 10 kHz ultrasonic transducer was small, and the reflected echo from the container wall interfered with the calibration of the ultrasonic transducer, thereby resulting in inaccurate measurement results, as shown in Table 2.

**Figure 8:**
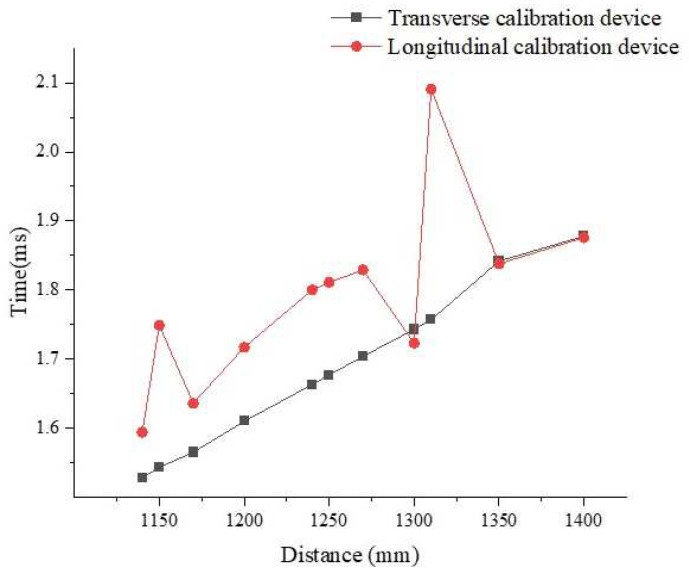
Comparison of 10 kHz ultrasonic transducer’s measurements with two types of ultrasonic-transducer calibration devices.

Comparing the calibration device of the transverse ultrasonic transducers with that of the longitudinal ultrasonic transducers for 10 kHz collected data shows that the calibration effect of the transverse-ultrasonic-transducer calibration device on the 10 kHz ultrasonic transducer is better than that of the longitudinal-ultrasonic-transducer calibration device, as shown in Figure 8. Because the diameter of the test cylinder used in the calibration devices shown in Figure 6 is small, the wall of the test cylinder also reflects echo, and different heights cause different degrees of interference to the ultrasonic transducer, resulting in certain nonlinear errors and affecting the calibration accuracy. Therefore, the calibration ultrasonic transducer finally selects the calibration platform for the transverse ultrasonic transducer error for calibration.

**Figure 9:**
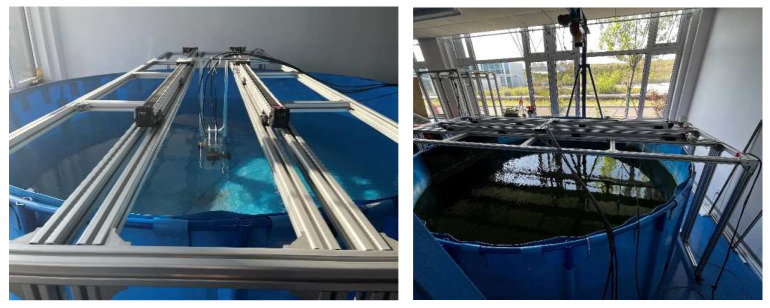
Error calibration platform for the transverse ultrasonic transducer.

The horizontal calibration platform consisted of a horizontal ultrasonic-transducer calibration device, two ultrasonic transducers, an STM32-based time-data acquisition module, and a computer system. Figure 9 shows that the horizontal calibration platform has a large measurement distance and is suitable for low-frequency ultrasonic transducers. To increase the measurement accuracy, a horizontal calibration platform was used to calibrate the 10 kHz ultrasonic transducer in this experiment.

**Figure 10:**
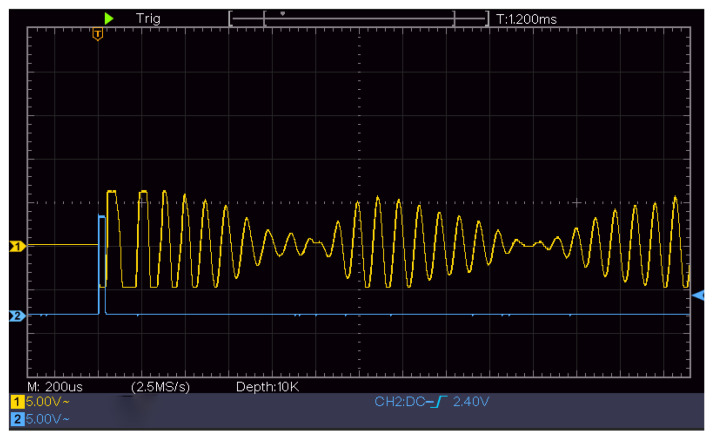
Negative half-wave representing first echo of ultrasonic transducer.

The range characteristic of the ULISSD consists of calculating the first positive half-wave of the first echo, as shown in Figure 10. The falling edge waveform is the ranging waveform of the ultrasonic logging tool, and the distance of the falling edge is calculated from the time when the ultrasonic probe emits the wave to when the echo is received. The ranging waveform locks the first positive half-wave of the first echo.

According to the varying nature of ultrasonic transducers, the first waveform of the echo of some ultrasonic transducers is a negative half-wave. Because the negative half-wave is filtered out after processing by the in-hole circuit of the ultrasonic logging tool, the waveform of the half cycle is less than that of the measured callback time compared with the actual echo time, as shown in Figure 10. To ensure the accuracy of the measured time, the measurement time of the ultrasonic transducer should be compensated by a half-cycle time during calibration.

### 2.8. Ultrasonic Transducer Calibration Steps

**Figure 11:**
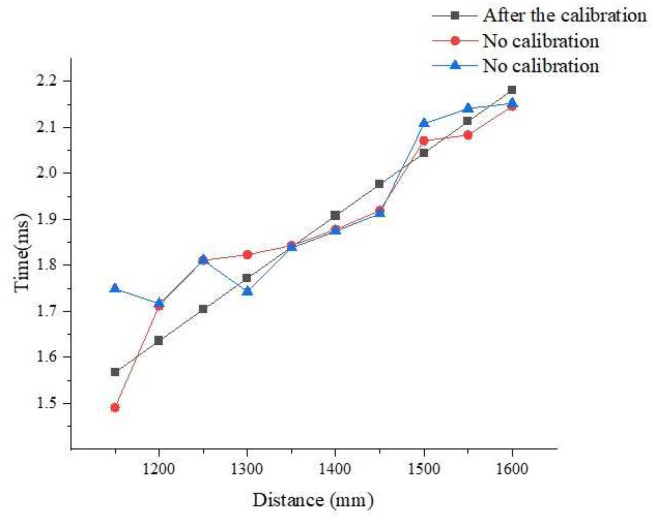
Comparison of data of transducer No. 1 before and after calibration.

The experiment was conducted according to the abovementioned method. Because the frequency of the ultrasonic transducer used by the ultrasonic logging tool was 10 kHz, the ultrasonic transducer was not suitable as a calibration device for high-frequency ultrasonic transducers, as shown in Figure 8. The high-frequency ultrasonic-transducer calibration device shown in Figure 11 was the calibration device used in the ultrasonic logging instrument laboratory of the Research Institute of Detection and Control of Qilu University of Technology (Shandong Academy of Sciences). The measurement distance of this calibration device was higher than that of the high-frequency ultrasonic-transducer calibration device shown in Figure 6. The experimental steps were as follows:

1. Connect the transducer to the calibration unit.
2. Determine the initial distance of the transducer from the reflecting surface.
3. Start the ULISSD and calibration device to read the time of flight (TOF) at the initial distance.
4. Adjust the distance, *D_x_*, several times and record the corresponding TOF, *t_x_*.
5. Import the measured data into Equation (4).
6. Calculate *D*_0_ and the ultrasonic propagation velocity, *v*, in the medium.

## 3. Results

### 3.1. Experimental Data

To ensure the reliability of the results of the experimental data, 10 iterations of an independent experiment were repeated based on the original height of 1350 mm. Each probe height was adjusted by 50 mm; a probe then recorded the received echo time; and finally, a probe at the bottom fixed distance, *D_x_*, obtained the echo time, *t_x_*. The acoustic propagation velocity, *v*, in water was calculated. The values are presented in Table 3.

By importing the data in Table 3 into Equation (4), we concluded that the distance error, *D*_0_, of ultrasonic transducer No. 1 was 1.47 mm, and the propagation speed of the ultrasonic wave in the medium during the experiment was 1467 m/s.

To ensure the rigor of the experimental conclusions, the same steps were performed for the second ultrasonic transducer of the horizontal calibration device. The recorded experimental data are presented in Table 4.

By using the previous calculations, the *D*_0_ for probe No. 2 was 1.49 mm, and the *v* was 1493 m/s. The difference in the transmission speed of the ultrasonic wave from that of probe No. 1 was attributed to the temperature during the experiment.

Because each ultrasonic transducer had certain differences, the distance between the piezoelectric plate of the ultrasonic transducer and the surface varied, and the influence of this distance led to different sound velocities during the test. The two ultrasonic-transducer-calibration experiments were conducted in an environment of 20 °C (±2). The experimental temperatures were different before and after, and different sound velocities were recorded at the end. However, the same temperature was guaranteed when testing the same ultrasonic transducer. Therefore, the calibration of the ultrasonic transducer was not influenced.

The feasibilities of the fixed-error and error-correction principles proposed in this study were verified by the aforementioned experiments and data. Owing to the varying nature of the transducers, the fixed error of each transducer was also different. To further improve the accuracy of the ultrasonic logging instrument developed in this study, four ultrasonic transducers of each product were calibrated individually to ensure that both the diameter value and offset value measured in engineering applications were more accurate.

The calibration principle, calibration platform, and experimental method described in this study are used to calculate the distance between the piezoelectric plate of the ultrasonic transducer and the transducer surface to reduce the error and improve the accuracy of the ULISSD in large-aperture logging. The calculated sound velocity serves only as a reference.

### 3.2. Comparison of Experimental Results

To verify the results of the ultrasonic transducer’s calibration, the experimental data before and after the calibration of ultrasonic transducers No. 1 and No. 2 were compared and analyzed.

Figure 11 shows a comparison of the data before and after the calibration of ultrasonic transducer No. 1. Before the calibration, the values measured by the ultrasonic logging instrument were scattered, and the measured distances were not stable.

**Figure 12:**
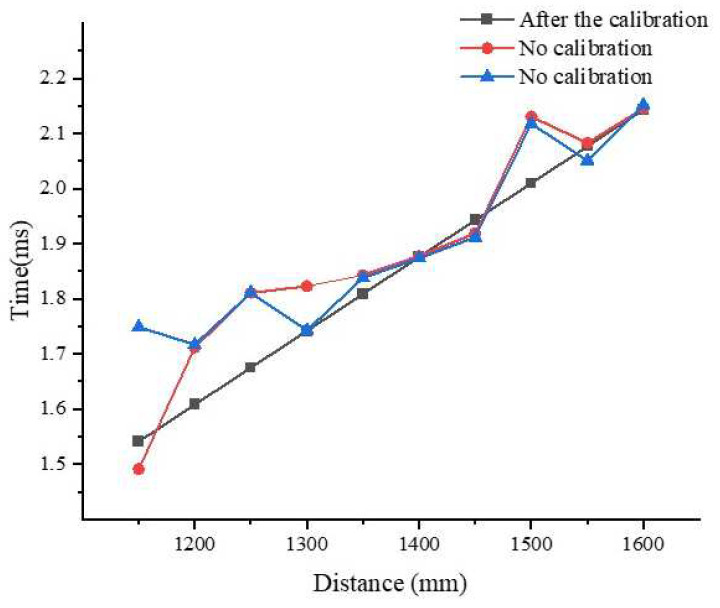
Comparison of data of transducer No. 2 before and after calibration.

The comparison of the data before and after the calibration of transducer No. 2 is similar to that of No. 1, as shown in Figure 12.

The comparison of both ultrasonic transducers with two sets of data suggests that the calibrated ultrasonic transducers can improve the measurement accuracy and reliability of the ULISSD to some extent.

## 4. Conclusions

In this study, we developed a device based on the error of an ultrasonic-transducer calibration platform. We also proposed a calibration method for an ultrasonic transducer which was successfully applied to the ULISSD, using a central-air-return-shaft-drilling technique based on the ultrasonic-transducer-calibration data. In the experiment, the ultrasonic-transducer calibration device could measure the distance from the piezoelectric transducer sheet to the surface of the ultrasonic transducer. In the process of the ULISSD logging, the distance from the piezoelectric sheet to the surface of the ultrasonic transducer could be subtracted from the calculation to obtain a more accurate diameter value. This result can improve the accuracy of future coal-mine-excavation works, facilitate the calculation of the drilling offset, and reduce drilling costs. The comparison before and after the application validated the effectiveness of this method. The device algorithm was extremely simple, and the cost of building the platform was extremely low; thus, the calibration of ultrasonic transducers can satisfy the actual needs in the field of drilling technology with minimal effort. The calibration method and calibration device described in this study are also suitable for ultrasonic ranging in other media.

## Figures and Tables

**Table 1:** Primary parameters of probe-error-correction platform.

Parameters	Numerical Value
Height	2350–2800 mm
Probe-holder adjustable distance	0–1100 mm
Medium	Water

**Table 2:** Test data of 10 kHz ultrasonic transducer on the longitudinal-transducer calibration platform.

Distance (mm)	Time (μs)	Speed (m/s)
1310	2091	1253
1300	2090	1244
1270	1829	1389
1240	1800	1378
1220	1781	1370
1200	1749	1373
1170	1636	1430
1140	1594	1430
970	1284	1511
920	1218	1511

**Table 3:** Time and acoustic propagation velocity of the echo received by probe No. 1 at different distances.

Distance (mm)	Time (μs)	Velocity (m/s)
1350	1838	1467
1400	1906	1467
1450	1974	1467
1500	2042	1467
1550	2110	1467
1600	2178	1467
1300	1770	1467
1250	1702	1467
1200	1634	1467
1150	1566	1467

**Table 4:** Time and acoustic propagation speed of the echo received by probe No. 2 at different distances.

Distance (mm)	Time (μs)	Velocity (m/s)
1350	1811	1493
1400	1878	1493
1450	1945	1493
1500	2012	1493
1600	2146	1493
1550	2079	1493
1300	1744	1493
1250	1677	1493
1200	1610	1493
1150	1543	1493
